# New Screening System Using Forward-Viewing Radial Endoscopic Ultrasound and Magnetic Resonance Imaging for High-Risk Individuals With Familial History of Pancreatic Cancer

**DOI:** 10.3389/fmed.2022.928182

**Published:** 2022-06-28

**Authors:** Reiko Ashida, Tatsuya Ioka, Ryoji Takada, Nobuyasu Fukutake, Kenji Ikezawa, Kazuyoshi Ohkawa, Shigenori Nagata, Hidenori Takahashi

**Affiliations:** ^1^Second Department of Internal Medicine, Wakayama Medical University, Wakayama, Japan; ^2^Department of Cancer Survey and Gastrointestinal Oncology, Osaka International Cancer Institute, Osaka, Japan; ^3^Department of Gastroenterological, Breast and Endocrine Surgery, Yamaguchi University Graduate School of Medicine, Yamaguchi, Japan; ^4^Department of Hepatobiliary and Pancreatic Oncology, Osaka International Cancer Institute, Osaka, Japan; ^5^Department of Gastroenterology, Osaka National Hospital, Osaka, Japan; ^6^Department of Diagnostic Pathology and Cytology, Osaka International Cancer Institute, Osaka, Japan; ^7^Department of Surgery, Osaka International Cancer Institute, Osaka, Japan; ^8^Department of Gastroenterological Surgery, Graduate School of Medicine, Osaka University, Osaka, Japan

**Keywords:** EUS, pancreatic cancer, family history, early chronic pancreatitis, IPMN

## Abstract

**Background and Aims:**

Attention is increasingly being paid to family history of pancreatic cancer (PC) as a risk factor for developing PC. It is mandatory to develop a screening system for early detection of PC; however, the relationship between a family history of PC and the incidence of pancreatic abnormalities, such as pancreatic cyst and chronic pancreatitis (CP), in the Japanese population remains unknown.

**Patients and Methods:**

Individuals with a family history of PC were prospectively enrolled in a screening program using forward-viewing radial endoscopic ultrasound (FR-EUS) and magnetic resonance imaging (MRI) with magnetic resonance cholangiopancreatography (MRCP) as the diagnostic modalities.

**Results:**

In total, forty-three individuals in 37 families were enrolled (mean age, 54 years). All individuals underwent FR-EUS and MRI with no complications. FR-EUS revealed resectable PC (*n* = 1, 2.3%), pancreatic cysts (*n* = 24, 55.8%), intraductal papillary mucinous neoplasm (IPMN; *n* = 13, 30.2%), and early CP-like appearance (*n* = 15, 34.9%). The detection rate of early CP-like appearance was significantly higher by EUS than by MRI. Pancreatic cysts and IPMN detected by FR-EUS were significantly correlated to age (≥60 years) and less correlated to men (hazard ratio [HR] 22.4; 95% confidence interval [CI], 2.10–236.0; *p* < 0.01 and HR 0.092; 95% CI, 0.01–0.83; *p* = 0.033, respectively). Early CP-like appearance detected by FR-EUS was significantly correlated with men and smoking (HR 5.0; 95% CI, 1.3–19.3; *p* = 0.02 and HR 4.02; 95% CI, 0.991–16.3; *p* = 0.05, respectively).

**Conclusion:**

A screening system using FR-EUS and MRI/MRCP for individuals with a family history of PC was useful for identifying curable PC and pancreatic abnormalities. The incidence of pancreatic cysts, such as IPMN and early CP-like appearance, was also high in the Japanese cohort.

## Introduction

Pancreatic cancer (PC) is now the fourth leading cause of cancer mortality in Japan (1). The incidence of PC is continuously increasing; however, its prognosis remains poor (5-year survival rate < 10%) as more than 80% of cases are unresectable at diagnosis (2). Although it appears mandatory to develop an efficient screening system, mass screening for PC is not recommended due to the relatively low incidence of sporadic PC. Therefore, the selection of screening targets is important.

Comprehensive genome profiling has recently become available, and many genes related to PC have gradually been elucidated (3). It has also become clear that approximately 5–10% of PCs are inherited and that the risk of developing PC is increased in several hereditary syndromes, such as hereditary breast ovarian cancer syndrome (HBOC), Lynch syndrome, and hereditary pancreatitis (4–7).

Strong familial history also increases the risk of PC. Familial pancreatic cancer (FPC) is defined as the occurrence of PC in two or more first-degree relatives that do not fulfill the criteria of other hereditary cancer syndromes according to the 2006 Pancreatic Cancer Genetic Epidemiology Consortium (PACGENE) (8). As FPC increases the risk of PC by 4–32 times, screening has been recommended for these high-risk individuals (HRIs) to detect PC at the early stage (9). Although the utility of such programs has been reported in the US and Europe since the early 2000s (10–12), these screening programs have not yet been applied internationally and data from Asian countries, such as Japan, are still lacking.

Although the benefit of pancreatic screening in terms of curative potential remains unknown and recommended frequency of examination during surveillance has not been determined to detect a malignant lesion in the early stage (13), expert panels have recommended endoscopic ultrasound (EUS) and magnetic resonance imaging (MRI) as suitable screening modalities (14, 15). Of these, EUS is limited due to its oblique view that requires high endoscopic skill and prevents visualization of the entire gastrointestinal (GI) tract. On the other hand, forward-viewing radial EUS (FR-EUS) enables the scope to be introduced with direct vision and to perform whole GI screening and pancreato-biliary screening simultaneously. As HRIs with a background of familial PC may also have a potential to develop GI cancer, screening of HRIs using FR-EUS and MRI could be a useful strategy for detecting curable cancer while the patient is asymptomatic.

Therefore, the aim of the study was to clarify the Japanese incidence of pancreatic tumor and high-risk abnormalities for developing PC, such as intraductal papillary mucinous neoplasm (IPMN) (16) and chronic pancreatitis (CP) (17), in individuals with a family history of PC by using FR-EUS and MRI.

## Materials and Methods

### Patient Selection and Study Design

This prospective study was conducted at a tertiary cancer institution in Japan between March 2016 and February 2020. The study protocol was approved by the Osaka International Cancer Institution (OICI) Institutional Review Board (IRB no. 1512046243), and written informed consent was obtained from all participants before each procedure.

The inclusion criteria were a family history of pathologically diagnosed pancreatic adenocarcinoma within a second-degree relative and age 20–75 years at the time of registration. Exclusion criteria were claustrophobia and the surgical history of total gastrectomy (due to insufficient examination by EUS). The individuals who already had symptoms, such as abdominal pain, or were previously diagnosed with pancreatic diseases, such as pancreatic cyst, were excluded.

The primary end point of the study was the prevalence of pancreatic diseases, such as PC and pancreatic endocrine tumor. Secondary end points were the frequency of high-risk factors for PC, such as pancreatic cysts, dilation of the main pancreatic duct (MPD), IPMN, and early CP-like appearance.

The current study protocol is a single examination using EUS and MRI as a screening modality. The individuals who were diagnosed with pancreatic diseases, such as pancreatic cysts, were followed in general clinical setting.

### Endoscopic Ultrasonography

**FIGURE 1:**
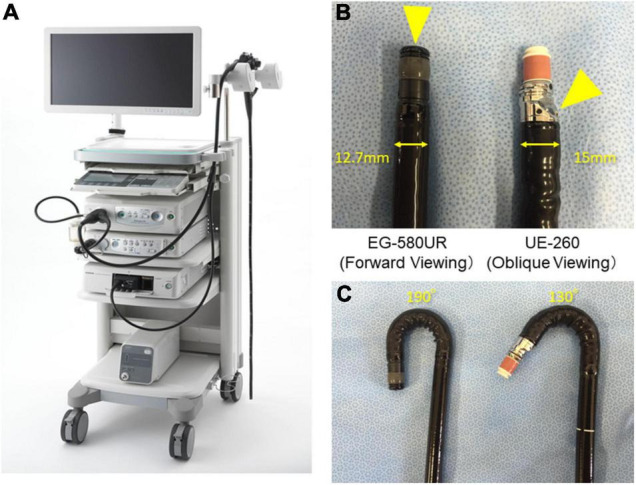
Endoscopic ultrasound (EUS) system and differences between forward-viewing radial EUS (FR-EUS) and conventional radial EUS. **(A)** EG-580UR with SU-1 EUS System (Fuji Medical, Tokyo, Japan). **(B)** The optical lens is located at the front in FR-EUS but behind the ultrasound transducer in conventional radial EUS. **(C)** The degree of up angle of FR-EUS is 190° but 130° in conventional radial EUS.

Endoscopic ultrasound was performed by a single experienced endosonographer (RA) who had performed diagnostic EUS examinations over 15 years. Screening of the pancreas was performed using an FR-EUS scope (EG-580UR) with an SU-1 EUS System (Fuji Medical, Tokyo, Japan; Figure 1A) at 7.5 MHz using the water-filled balloon technique after intravenous conscious sedation. EG-580UR has a thinner diameter of 12.7 mm than conventional EUS and max up angulation is 190°, which allows precise screening in the stomach (Figures 1B,C).

**FIGURE 2:**
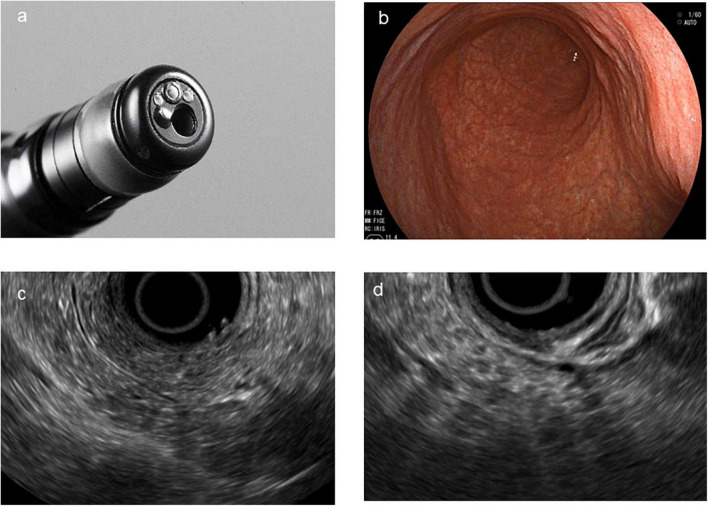
Direct forward-viewing radial endoscopic ultrasound (FR-EUS). **(a)** The image of the tip of FR-EUS. **(b)** Endoscopic view of the stomach. **(c)** EUS image showing echogenic foci, echogenic strands, and echogenic ductal walls. **(d)** EUS image showing typical lobularity.

The pancreatic parenchyma and pancreatic duct were assessed to change the characteristics of CP using the Japanese guidelines for CP. All pancreatic parenchyma tissues were assessed for the presence of echogenic foci, echogenic strands, lobularity, cysts, and calcification. The diameter of the MPD was measured in the head, body (at the level of the portal vein–superior mesenteric vein confluence), and tail. The pancreas was also evaluated for hyperechoic ductal margin, irregularity of the MPD, and visible dilated side branches. Early CP was defined as the presence of finding (1) or finding (2) and at least one additional EUS finding, as follows: (1) echogenic foci/echogenic strands, (2) lobularity, (3) echogenic ductal walls, and (4) dilated side branches (Figure 2).

### Magnetic Resonance Imaging and Magnetic Resonance Cholangiopancreatography

All MRI images were obtained at 1.5 T (HD EXCITE; GE Healthcare Japan, Tokyo, Japan) using the body coil for signal transmission and an eight-channel body array coil for the reception. Abdominal organs were localized on T2-weighted single-shot fast spin-echo images obtained in the three cardinal planes.

The pancreatic duct was imaged on three-dimensional magnetic resonance cholangiopancreatography (MRCP) that included the entire pancreas and duodenum. DWI sequences covering the liver and pancreas were performed at low (50 s/mm^2^) and high (1,000 s/mm^2^) b-values with respiratory gating. Apparent diffusion coefficient (ADC) maps were automatically calculated by monoexponential fitting.

According to the Japanese clinical diagnostic criteria for CP (2019), irregular dilation of three or more branched pancreatic ducts on MRCP suggests early CP. All images were analyzed by several radiologists who had at least 10 years of experience in pancreatic imaging and were blinded to patients’ risk factors for PC.

### Statistical Analysis

The significance of differences in continuous data was assessed with respect to the background data using paired or non-paired Student’s *t*-tests as a reference. The chi-squared test was used to analyze qualitative data. Univariate and multivariate analyses were performed to identify variables significantly associated with the detection of pancreatic cysts and early CP. After calculating the hazard ratio (HR) and 95% confidence interval (CI), multivariate analysis was performed using factors that were significant in univariate analysis to identify independent factors associated with pancreatic cyst and early CP. *p*-values < 0.05 were considered statistically significant. Statistical analysis was performed using JMP Pro ver. 14 (SAS Institute Japan Inc., Tokyo, Japan).

## Results

### Patients and Characteristics

The patient characteristics are listed in Table 1. A total of 43 individuals (18 men, 25 women; mean age, 54 years; age range, 32–75 years) from 37 families with a family history of pathologically confirmed pancreatic adenocarcinoma were referred to this screening system.

**TABLE 1:** Characteristics of high-risk individuals undergoing screening.

	*N* = 43 (%)
Mean age (y) (range)	54 (32–75)
Male: Female	18:25
Ever smokers	12 (27.9)
Current smokers	1 (2.3)
Heavy smokers*	4 (9.3)
Regular alcohol intake**	18 (41.8)
History of diabetes mellitus	0 (0)
High amylase (14-42 U/L)	8 (18.6)

*Smoking score = number × years > 500 number × years.

**Regular alcohol intake (≥2 drinks/week for women, ≥3 drinks/week for men).

Of the 43 individuals, 12 (27.9%) had a history of smoking, 1 (2.3%) was a current smoker at the time of screening, and 4 (9.3%) were heavy smokers with a smoking score (cigarette number × years) of 500 or more. In addition, 18 (41.8%) had a history of habitual alcohol drinking, defined as the consumption of alcohol 2 days/week for women and 3 days/week or more for men. None had diabetes. Blood tests revealed pancreatic hyperamylasemia (>42 U/L) in eight patients (18.6%).

Among first-degree relatives, there were 30 (69.8%) individuals with one family history of PC, 11 (25.6%) with two, and 1 (2.3%) with three. Among second-degree relatives, there were 16 (37.2%) with one family history of PC, 23 (53.4%) with two, and 4 (9.3%) with three. Among those with a family history of PC, 19 (44.2%) had a family history of young-onset PC (age at diagnosis ≤ 60 years). In addition, six had a cancer history other than PC, i.e., gastric cancer (*n* = 1), colon cancer (*n* = 1), esophageal cancer (*n* = 1), cervical cancer (*n* = 1), thyroid cancer (*n* = 1), and testicular cancer (*n* = 1; Table 2).

**TABLE 2:** Family history of pancreatic cancer and personal history of any cancer.

Risk group	*N* = 43 (%)
**Affected first-degree relatives with PC**	
0	1 (2.3)
1	30 (69.8)
2	11 (25.6)
3	1 (2.3)
**Affected family member with PC (within second-degree**)	
1	16 (37.2)
2	23 (53.4)
3	4 (9.3)
From a kindred with young-onset PC (age at diagnosis ≦ 60 years)	19 (44.2)
Personal history of cancer	6 (13.9)
Gastric cancer	1 (2.3)
Colon cancer	1 (2.3)
Esophageal cancer	1 (2.3)
Cervical cancer	1 (2.3)
Thyroid cancer	1 (2.3)
Testicular cancer	1 (2.3)

*PC, pancreatic cancer, first-degree relative: parent, child, and sibling.*

### Detection Rates of Pancreatic Tumor

The imaging features at the screening by EUS and MRI are summarized in Table 3. Of the 43 individuals who were screened, PC was detected in one individual (2.3%), by EUS. No pancreatic mass was detected on any MRI sequence, such as DWI. MRCP of the patients with PC showed disruption of the MPD and nearby cysts.

**TABLE 3:** Pancreatic features detected in screening by endoscopic ultrasound (EUS) and magnetic resonance imaging (MRI).

	*N* = 43 (%)
**Pancreatic lesions on EUS**	
Mass	1 (2.3)
Cyst/IPMN	24 (55.8)
MPD dilation	5 (11.6)
Early chronic pancreatitis	15 (34.9)
**Features of EUS**	
Echogenic foci	16 (37.2)
Echogenic strands	12 (27.9)
Lobularity	6 (14.0)
Echogenic duct walls	22 (51.2)
Visible side branches	17 (39.5)
Cysts	24 (55.8)
Calcification	0 (0.00)
**Features of MRI**	
Mass	0 (0.0)
Cyst/IPMN	22 (51.2)
MPD dilation	2 (4.7)

### Detection Rate of Pancreatic Cyst, Intraductal Papillary Mucinous Neoplasm, Main Pancreatic Duct Dilation, and Chronic Pancreatitis

Table 4 lists the pancreatic findings detected by EUS and MRI. There was no significant difference between EUS and MRI/MRCP for the detection of pancreatic cysts, which were found in 24 individuals (55.8%) by EUS and in 22 individuals (51.2%) by MRI/MRCP. Among individuals with cysts detected by EUS, a diagnosis of IPMN was made in 13 (30.2% of screened cases).

**TABLE 4:** Pancreatic findings according to modality.

	EUS	MRI	*P*-value
Pancreatic cancer	1 (32–75)	0 (0)	N.S
Pancreatic cyst	24 (55.8)	22 (51.1)	N.S
MPD dilation	5 (11.6)	1 (2.3)	0.202
Early chronic pancreatitis	15 (34.9)	0 (0)	<0.001

Main pancreatic duct dilation (>3.0 mm in pancreatic head and >2.5 mm in pancreatic body) was detected in five cases (11.6%) by EUS. Of these, the most dilated case (pancreatic body, 4.36 mm) was diagnosed as PC, and three with dilated MPD also had pancreatic cysts, compatible with IPMN. The remaining case of MPD dilation without pancreatic cysts showed an early CP-like appearance, although this individual reported no habitual alcohol use.

Magnetic resonance cholangiopancreatography found no early CP-like findings in any individual, whereas EUS detected early CP-like features in 15 individuals (34.9%): echogenic foci in 16 (37.2%), echogenic strands in 12 (27.9%), lobularity in six, echogenic ductal walls in 22 (51.2%), and visible side branches in 17 (39.5%; Table 2). The detection rate of early CP-like findings was significantly higher by EUS when compared with MRI (Table 4).

### Analysis of Factors Related to the Incidence of Pancreatic Intraductal Papillary Mucinous Neoplasm and Chronic Pancreatitis

Pancreatic cysts were found in 24 patients (55.8%) by EUS and were observed more frequently in those aged ≥ 60 years (<0.01; 95% CI, 2.10–236.0) and less frequently in male patients (*p* = 0.033; 95% CI, 0.01–0.83; multivariate analysis; Table 5).

**TABLE 5:** Results of analysis of factors related to a pancreatic cyst.

	Univariate analysis			Multivariate analysis		
		
	HR	95% CI	*p*	HR	95% CI	*p*
Age (≧60 years old)	6.3	(1.45–27.5)	0.014	22.4	(2.10–236.0)	<0.01
Sex (male)	0.45	(0.13–1.55)	0.206	0.092	(0.01–0.83)	0.033
Smoking	0.72	(0.19–2.75)	0.633			
Alcohol intake	0.982	(0.29–3.33)	0.977			
Cancer history	1.70	(0.276–10.5)	0.567			
Familial history of PC (more than 2)	1.87	(0.466–7.54)	0.376			

Univariate analysis revealed a significant association between the male sex (*p* = 0.02; 95% CI, 1.3–19.3) and history of smoking (*p* = 0.05; 95% CI, 0.991–16.3) with early CP (HR, 5.0 and 4.02, respectively; Table 6).

**TABLE 6:** Results of analysis of factors related to the early chronic pancreatitis.

	Univariate analysis			Multivariate analysis		
		
	HR	95% CI	*p*	HR	95% CI	*p*
Age (≧60 years old)	2.86	(0.775–10.5)	0.115			
Sex (male)	5.0	(1.3–19.3)	0.02	3.71	(0.87–15.8)	0.076
Smoking	4.02	(0.991–16.3)	0.05	2.41	(0.51–11.3)	0.263
Alcohol intake	0.889	(0.248–3.18)	0.856			
Cancer history	0.923	(0.149–5.74)	0.932			
Familial history of PC (more than 2)	0.909	(0.222–3.72)	0.894			

### Adverse Events

No procedure-related compilation was reported.

## Case Presentation

**FIGURE 3:**
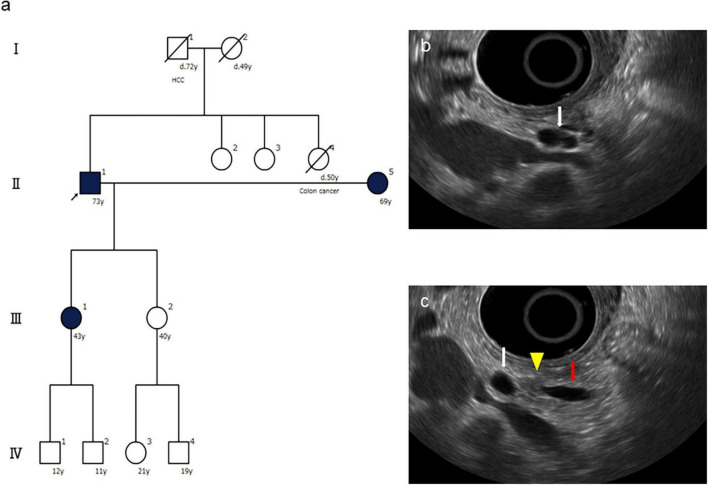
Family tree and endoscopic ultrasound (EUS) findings of the presented case. **(a)** The family tree shows one affected first-degree relative with pancreatic cancer (PC), an high-risk individual (HRI) (arrow) who was diagnosed with PC by this screening. **(b)** FR-EUS shows a cystic lesion (white arrow) in the pancreatic body. **(c)** FR-EUS shows a hypoechoic mass (arrowhead) in the pancreatic body between a pancreatic cyst (white arrow) and dilated MPD (red arrow).

**FIGURE 4:**
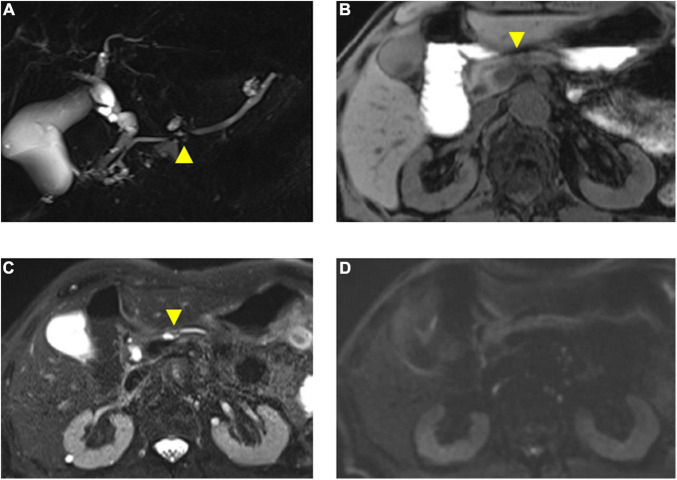
Magnetic resonance imaging findings of the presented case. **(A)** Magnetic resonance cholangiopancreatography (MRCP) shows disruption of the main pancreatic duct (MPD) (arrowhead) and a nearby cyst. **(B)** T1-weighted fat saturation MRI shows necking of pancreas parenchyma (arrowhead) with weak low-intensity change in a pancreatic body. **(C)** T2-weighted MRI shows dilated pancreatic duct and nearby cyst (arrowhead). **(D)** Diffusion-weighted MRI (*b* = 1,000) shows no sign of malignancy.

**FIGURE 5:**
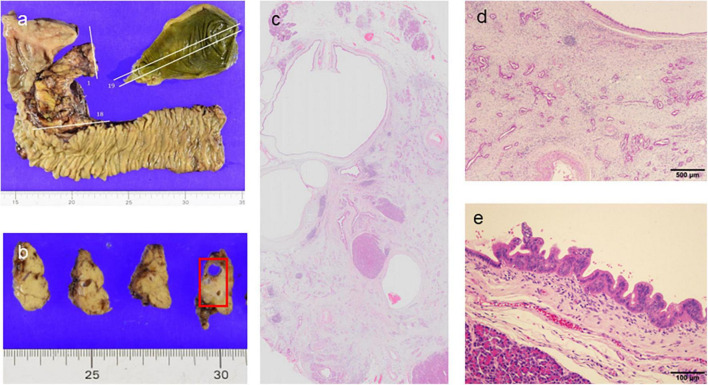
Pathological findings of the pancreato-duodenostomy specimen. **(a,b)** Macroscopic findings. **(c)** Loupe view showing ductal adenocarcinoma concomitant with high-grade intraductal papillary mucinous neoplasm (IPMN) (hematoxylin and eosin [HE] stain). **(d)** Moderately-differentiated ductal adenocarcinoma with fibrous stroma (HE, ×40). **(e)** Intraepithelial carcinoma arising in the IPMN component (HE, ×200).

A 73-year-old male was referred to the screening program after his daughter was diagnosed with PC at the age of 42 years. His wife was also diagnosed with PC, at the same time as their daughter was receiving chemotherapy (Figure 3a). He was a heavy smoker (smoking score, 1,080) and habitual drinker. His blood amylase levels were increased (total Amy, 181 [44–132 U/L]; p-Amy, 71 [14–42 U/L]). MRI/MRCP revealed disruption of the MPD and a nearby cyst, with dilation of the MPD on the distal side of the disruption (Figure 4). The FR-EUS findings were stenosis of the MPD in the body of the pancreas with a nearby cyst (Figure 3b), a 7 mm hypoechoic mass at the site of stenosis, and distal pancreatic duct dilation (Figure 3c). The background pancreatic parenchyma showed early CP and a multilocular cyst in the pancreatic head suggested the coexistence of branched type IPMN. ERCP was performed and pancreatic juice cytology was positive for adenocarcinoma. At this point, he refused surgery because he did not want to bother his daughter and wife who were both receiving chemotherapy at the time of his diagnosis. CT obtained 3 months later showed that tumor size had increased to 10 mm. The patient finally underwent pancreato-duodenostomy in response to persuasion by his daughter and wife. Pathological evaluation revealed invasive ductal adenocarcinoma, well-to-moderately differentiated type, pT1c, pN0, and M0: Stage IA (Figures 5a–d). Non-invasive IPMN-derived carcinoma was also found in the pancreatic head, measuring 45 mm (pTS3), pTis, pN0, M0: Stage 0 (Figure 5e).

Screening performed for his younger daughter, who had three affected first-degree relatives with PC, found no abnormalities in the pancreatic parenchyma or pancreatic duct. This daughter had no history of smoking or drinking, whereas the daughter who developed PC had a history of smoking when younger.

## Discussion

Since Klein et al. (9) estimated that the incidence of PC was increased according to the number of first-degree family members with PC (4.5–32 times), a family history of PC has been considered as a risk factor for developing PC. International Cancer of Pancreas Screening (CAPS) Consortium was then established with the aim of early diagnosis of PC for those with familial risk, and the first consensus conference was held in 2011 (14) with a recent update in 2019 (15). The consensus meeting strongly recommended EUS and MRI as screening modalities due to their high resolution and lack of ionizing radiation although a recent meta-analysis suggested the difficulty of diagnosis of malignant lesions in the early stage (18). Although several studies have reported the usefulness of screening in HRIs (10–12), these data had been published mainly from Western countries, and data from Asia, such as Japan, are still lacking. Therefore, we conducted the first screening study for HRIs in Japan with a familial history of PC, using FR-EUS and MRI.

Of the HRIs who participated in the study, 11 (25.6%) had two affected first-degree relatives and 1 individual (2.3%) had three affected first-degree relatives; therefore, only 12 of the 43 individuals (27.9%) fit the definition of FPC. However, within second-degree relatives, 27 of the 43 individuals (62.8%) had more than two affected relatives with PC, and 19 individuals (44.2%) had a family history of young-onset PC (≤60 years), suggesting that the present sample was a relatively high-risk population.

Of the present findings, the high incidence of pancreatic cysts and early CP-like features are noteworthy. Pancreatic cysts were observed in 24 of the 43 (55.8%) (mean age, 59.8 years; range, 42–73 years), which is higher than in the previously reported Japanese data, with a prevalence of 13.7% in the general population (19). Among individuals with pancreatic cysts, IPMN (which is obviously connected to the pancreatic duct) was observed in 13 of the 43 cases (30.2%) and the prevalence of pancreatic cysts that included IPMN was significantly correlated with age ≥60 years and less frequently in men (HR, 22.4; 95% CI, 2.10–236.0; *p* < 0.01 and HR, 0.092; 95% CI, 0.01–0.83; *p* = 0.033, respectively).

In contrast, univariate analysis showed that early CP-like appearance was significantly correlated with men and with smoking (HR, 5.0; 95% CI, 1.3–19.3; *p* = 0.02 and HR, 4.02; 95% CI, 0.991–16.3; *p* = 0.05, respectively). These results are similar to those of a previous screening study for individuals with familial history of PC (11) that suggested a relationship between epigenetic factors, such as smoking cigarettes, which may accelerate gene damage. Indeed, the present patient in whom PC was detected was a heavy smoker, and his daughter who developed PC also had a smoking history. His other daughter, who had no specific findings in the pancreas, had no history of smoking but did have a high-risk genetic background of three affected first-degree relatives with PC although the genetic analysis was not conducted in this family.

Regarding the choice of screening modality, EUS enables visualization of the pancreas at high resolution regardless of the subject’s body shape or GI gas condition (20). However, EUS is not in widespread use because it is difficult to maneuver, with oblique viewing and a large scope diameter. A direct FR-EUS with a thin diameter of 12.7 mm (EG-580UR; Fuji Medical, Tokyo, Japan) that was recently developed was used in the present study (Figure 1B). This scope also has a soft scope shaft and better flexibility as compared to conventional radial EUS, which allows precise screening in the stomach and safe maneuver during the EUS screening (Figure 1C). This scope reduces the technical difficulty of the procedure and therefore reduces the risk of the procedure; indeed, no procedure-related complications occurred in the present study. Therefore, FR-EUS maybe a suitable modality for repeat examination during surveillance for HRIs (21). It also enables simultaneous GI and pancreato-biliary screening. Although no cases of esophageal cancer or gastric cancer were found in this study cohort, given the possibility of a hereditary component, such as Lynch syndrome, EUS with direct forward viewing may be beneficial in terms of efficiency of screening and reducing procedure time, although further evaluation is required in the future study.

Magnetic resonance imaging with MRCP is also useful for objective visualization of the pancreatic duct and cysts, although the present results indicate that MRI/MRCP is less sensitive to EUS in terms of detecting early CP-like appearance, which may be improved by applying 3.0 T MRI. The indirect findings, such as ductal disruption, following dilated distal MPD and nearby pancreatic cyst were clearly detected by MRCP (Figure 4D) although the pancreatic mass was not detected even with DWI, which may be improved by a different sequence, such as DWI, with advanced post-processing and motion correction or intravoxel incoherent motion DWI (22, 23). Nevertheless, MRI/MRCP is a useful complementary imaging tool for PC screening that can detect small pancreatic cysts in the pancreatic tail or uncinate, which can be difficult to visualize by radial EUS. Therefore, combination screening using both FR-EUS and MRI/MRCP is recommended in the initial screening for HRIs.

Limitations of this study are the small number of participants and the single-arm study design. It will be necessary to conduct a future multi-center comparative study between individuals with a family history of PC and controls to prove the usefulness of this screening system.

## Conclusion

Screening using direct FR-EUS and MRI/MRCP for HRIs with a family history of PC appears to be safe and useful for the early detection of PC and pancreatic abnormalities. The incidence of pancreatic cysts, such as IPMN and early CP-like appearance, was also high in the Japanese cohort.

## Data Availability Statement

The raw data supporting the conclusions of this article will be made available by the authors, without undue reservation.

## Ethics Statement

The studies involving human participants were reviewed and approved by Osaka International Cancer Institution (OICI) Institutional Review Board (IRB No. 1512046243). The patients/participants provided their written informed consent to participate in this study.

## Author Contributions

RA and TI designed the research. RA performed screening and drafted the manuscript. RT, NF, KI, and KO performed the research and analyzed the data. SN evaluated the pathological assessment and drafted the manuscript. HT performed the surgery and a revision of the draft. All authors have read and agreed to the final manuscript.

## Conflict of Interest

TI received a speaker’s fee and consulting fees as an advisory role with Taiho Pharmaceutical. The remaining authors declare that the research was conducted in the absence of any commercial or financial relationships that could be construed as a potential conflict of interest.

## Publisher’s Note

All claims expressed in this article are solely those of the authors and do not necessarily represent those of their affiliated organizations, or those of the publisher, the editors and the reviewers. Any product that may be evaluated in this article, or claim that may be made by its manufacturer, is not guaranteed or endorsed by the publisher.
